# The minimal clinically important difference changes greatly based on the patient's baseline clinical status

**DOI:** 10.1002/jeo2.70137

**Published:** 2025-02-10

**Authors:** Marco Franceschini, Angelo Boffa, Alessandro Di Martino, Elettra Pignotti, Luca Andriolo, Stefano Zaffagnini, Giuseppe Filardo

**Affiliations:** ^1^ Università di Bologna Bologna Italy; ^2^ Applied and Translational Research (ATR) Center IRCCS Istituto Ortopedico Rizzoli Bologna Italy; ^3^ Clinica Ortopedica e Traumatologica 2, IRCCS Istituto Ortopedico Rizzoli Bologna Italy; ^4^ Faculty of Biomedical Sciences Università della Svizzera Italiana Lugano Switzerland

**Keywords:** baseline, clinical status, knee, MCID, osteoarthritis

## Abstract

**Purpose:**

To quantify the influence of baseline values of a specific patient‐reported outcome measure (PROM) on the minimal clinically important difference (MCID) calculation in a homogeneous series of knee osteoarthritis patients treated with platelet‐rich plasma (PRP) injections.

**Methods:**

A data set of 312 patients with knee osteoarthritis treated with intra‐articular PRP injections was used. Patients were evaluated through the International Knee Documentation Committee (IKDC) subjective score at 6 months after treatment. According to the baseline IKDC score, the study population was stratified into eight clusters in the first phase (<20, 20–29, 30–39, 40–49, 50–59, 60–69, 70–79 and ≥80) and in three macro clusters in the second phase (<40, 40–69 and ≥70). MCID for the IKDC score was calculated through an anchor‐based method in both phases.

**Results:**

The MCID calculation was performed for the eight clusters according to the baseline IKDC values, obtaining values from 16.2 to −3.1. Afterwards, further MCID calculation was performed after unifying patients in three major clusters based on the similarity of the previously obtained MCID values. Ninety‐six patients reported a baseline IKDC score <40, 173 patients between 40 and 70, and 43 patients ≥70. MCID values for the three macro clusters were: 14.6 for patients with baseline IKDC score <40, 7.2 for patients with values between 40 and 69, while patients with values ≥70 reported an MCID value of −2.8.

**Conclusions:**

This study demonstrated that the baseline patient clinical status influences the improvement needed to be perceived as clinically relevant. Patients with a worse baseline clinical status presented higher MCID levels, while MCID lost significance in patients with high baseline clinical values. These findings warrant applying general thresholds to a patients' cohort, showing the remarkable impact of the baseline clinical status. Patient stratification ensures a proper quantification of MCID values and the identification of patients benefiting from the studied treatment.

**Level of Evidence:**

Level 4.

**Abbreviations**
ADLs: activities of daily living
IKDC: International Knee Documentation Committee
KOOS: Knee Injury and Osteoarthritis Outcome Score
KL: Kellgren–Lawrence
MCID: minimal clinically important difference
OA: osteoarthritis
PROM: patient‐reported outcome measure
PRP: platelet‐rich plasma

## INTRODUCTION

The minimal clinically important difference (MCID) is a patient‐centred psychometric parameter that allows physicians to express both the statistical significance after a given treatment and the clinical benefit perceived by the patient [3]. MCID was originally defined as ‘the smallest difference in score in the domain of interest which patients perceive as beneficial and which would mandate, in the absence of troublesome side effects and excessive cost, a change in the patient's management’ [10]. This concept, first proposed by Jaeschke in 1989, is gaining popularity in the scientific literature as a metric for understanding and properly evaluating the results from clinical trials that use patient‐reported outcome measures (PROMs) [12]. Despite the increasing interest, there are still pitfalls and controversies, which prevent an effective and broader application of MCID when interpreting clinical results [6].

Different aspects should be considered when calculating MCID in relation to a specific PROM. Recent studies demonstrated that the different methods available to calculate MCID, generally classified as anchor‐based or distribution‐based, can lead to different values with high heterogeneity, which may affect the percentage of patients achieving the MCID level for the specific PROM investigated [8, 15, 17]. Among the different methods, the anchor‐based approach is emerging as the most suitable one [22], but this may not suffice to ensure a proper application of MCID. In fact, besides the methodological calculation side, another critical side is represented by the demographic characteristics of the population which may contribute to the MCID variability. Data, such as disease, treatment, and follow‐up, can significantly influence the MCID [11, 21]. Accordingly, specific MCID values have been calculated for specific treatments, diseases, and follow‐up times [2, 4, 14]. However, another characteristic has been suggested to affect MCID calculation, the baseline clinical status of the patients [20]. A full understanding of how this variable can affect MCID is necessary to give this psychometric parameter a more clinically relevant value.

The aim of this study was to quantify the influence of the baseline values of a PROM (the International Knee Documentation Committee [IKDC] subjective score) on the MCID threshold calculation in a homogeneous series of patients all affected by knee osteoarthritis (OA) and treated with platelet‐rich plasma (PRP) injections.

## MATERIALS AND METHODS

The data used to analyze the influence of the baseline clinical status on the MCID calculation were based on prospectively collected data set of knee OA patients treated with intra‐articular PRP injections between March 2009 and November 2020 (institutional review board approval Prot. no. 0015664) at the Rizzoli Orthopedic Institute (Bologna, Italy). Informed consent was obtained at the time of patients' enrolment. PRP treatment consisted of three intra‐articular injections at 1‐week intervals and was indicated in patients with unilateral symptomatic knee OA with a history of chronic pain (at least 6 months) or swelling, early OA findings at imaging evaluation with signs of cartilage degeneration (Kellgren–Lawrence [KL] Grade = 0, detected on magnetic resonance imaging) or OA (KL Grade = 1–4), and age between 18 and 80 years.

Patients were evaluated through the IKDC subjective score and the Knee Injury and Osteoarthritis Outcome Score (KOOS) at baseline and 6 months after the injective treatment. The IKDC subjective score baseline values were used to stratify the study population. According to Terluin et al. [20], a second PROM of interest was used to avoid biases in the analysis, the KOOS Activities of Daily Living (ADL) subscale. The Pearson correlation between the baseline value of the IKDC subjective score and the KOOS ADL subscale was calculated to support the scores' stratification [20]. At 6 months, patients were asked to express an overall opinion on the treatment received by answering an explicit anchor question, rating on a 6‐point scale their clinical condition compared to the baseline: ‘Compared with before the injective treatment, how would you rate your knee now?’ (1 *total recovery*; 2 *much better*; 3 *a little better*; 4 *no change*; 5 *a little worse*; 6 *much worse*).

A total of 312 patients (194 men and 118 women, mean age 53.6 ± 11.4 years, mean body mass index 26.7 ± 5.0 kg/m^2^) were included in this study. The study population was stratified according to the baseline IKDC subjective score in eight clusters (<20, 20–29, 30–39, 40–49, 50–59, 60–69, 70–79 and ≥80). In a second phase, the subgroups were collected in three macro clusters to finalize the analysis (patients with baseline IKDC subjective score <40, patients with baseline IKDC subjective score between 40 and 69, and patients with baseline IKDC subjective score ≥70). Using the collected clinical data of this series, MCID was calculated for the IKDC subjective score through an anchor‐based method as the mean observed change score in the ‘a little better’ group both in the first stage on eight clusters and in the second stage analysis on three macro clusters [8].

## RESULTS

The statistical analysis showed a high positive correlation between the baseline IKDC subjective score and the baseline KOOS ADL subscale (Pearson correlation coefficient = 0.744, *p* < 0.0005) [13]. Accordingly, the stratification of patients' population based on the IKDC subjective score baseline values could be considered acceptable according to Terluin et al. [20]. Thus, the study population was stratified in relation to the baseline value of the IKDC subjective score, as reported in Table 1. Ninety‐six patients reported a baseline IKDC subjective score <40, 173 patients between 40 and 70 and 43 patients ≥70.

**Table 1:** Patients' stratification based on the International Knee Documentation Committee (IKDC) subjective score baseline values.

IKDC subjective score	Clusters	Macro clusters
Baseline values	Patients (%)	Patients (%)
<20	12 (4%)	96 (31%)
20–29	33 (11%)	
30–39	51 (16%)	
40–49	76 (24%)	173 (55%)
50–59	62 (20%)	
60–69	35 (11%)	
70–79	31 (10%)	43 (14%)
≥80	12 (4%)	

The analysis of the answers given by the patients to the anchor question for the MCID calculation documented that thirteen (4.2%) patients reported that their knee was totally recovered, 147 (47.1%) patients reported that their knee was ‘much better’ than before treatment, 89 (28.5%) patients reported that their knee was ‘a little better’, 48 (15.4%) reported that they were about the same, 12 (3.8%) reported that they were ‘a little worse’ and 3 (1.0%) reported that their knee was ‘much worse’. The overall IKDC subjective score significantly improved from the basal value of 48.5 ± 16.7 to 62.3 ± 19.0 at 6 months of follow‐up (*p* < 0.0005).

**Figure 1:**
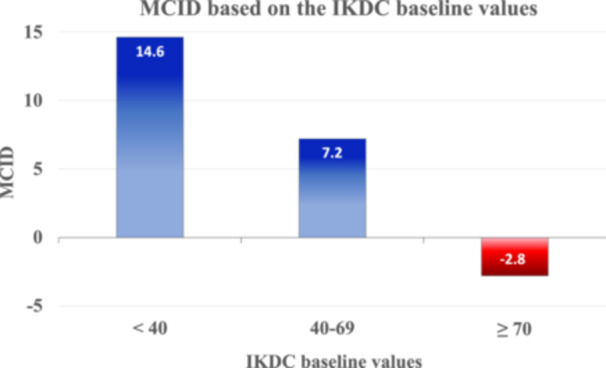
MCID values based on the International Knee Documentation Committee (IKDC) subjective score baseline values for each major cluster. MCID, minimal clinically important difference.

After the stratification of the population in eight clusters according to the baseline IKDC values, the MCID calculation was performed for each cluster, obtaining values from 16.2 to −3.1, as reported in Table 2. Afterwards, further MCID calculation was performed after unifying patients in three major clusters based on the similarity of the previously obtained MCID values. Accordingly, MCID values for the three macro clusters were 14.6 for patients with baseline IKDC subjective score <40, 7.2 for patients with values between 40 and 69, while patients with values ≥70 reported an MCID value of −2.8, as reported in Figure 1.

**Table 2:** MCID values obtained for the eight clusters and the three macro clusters based on the International Knee Documentation Committee (IKDC) subjective score baseline values.

IKDC subjective score	Clusters	Macro clusters
Baseline values	MCID values	MCID values
<20	14.4	14.6
20–29	16.2	
30–39	13.5	
40–49	8.1	7.2
50–59	6.0	
60–69	8.1	
70–79	−2.7	−2.8
≥80	−3.1	

Abbreviations: MCID, minimal clinically important difference.

## DISCUSSION

The main finding of this study is that the baseline patient clinical status influences the improvement needed to be perceived as clinically relevant. Patients with a worse baseline clinical status presented higher MCID levels, while MCID lost significance in patients with high baseline clinical values.

The MCID represents a key aspect of current clinical research, with a growing application in clinical studies over the years [5]. It was originally introduced over 30 years ago to better understand the change in questionnaire scores and to bridge the gap between statistical and clinical significance [10]. In fact, it has been proposed to better quantify the extent of the clinical benefit reported by patients after receiving a specific treatment for a given disease, beyond the statistical significance [4]. To date, MCID is more and more applied in the literature to analyze the results of clinical trials and better reflect the rate of treatment success [8]. Nevertheless, some critical aspects remain in terms of MCID calculation and interpretation, fostering further research for optimizing its application.

The methods available in the literature to calculate MCID have been recently reviewed and discussed by Bloom et al. [3]. The different calculation methods can be generally divided into anchor‐based and distribution‐based, each one with specific advantages and pitfalls. The anchor‐based methods determine the MCID in reference to an external subjective criterion used to evaluate the extent of the change in a PROM [9]. Unfortunately, external criteria and the selection or grouping of the different scale levels are themselves flawed, as they suffer from their own imperfections, given the limited standardization in their design and evaluation [3]. On the other hand, the distribution‐based methods are purely statistical approaches and are strictly related to patients' characteristics. However, these methods do not use clinical questionnaires and do not consider the patient's perspective in terms of clinically important change [18]. The calculation method choice is not a trivial aspect, as shown in a recent study underlying how different methods can lead to heterogeneous MCID values [8]. The use of a large number of anchor‐ and distribution‐based methods led to a wide range of values in the same population, making it difficult to estimate the real effectiveness of the performed treatment [8]. In this context, the most reliable calculation method and consequently its choice remains controversial, although there is an increasing agreement towards using more patient‐centred anchor‐based methods [1, 3, 21].

The use of an anchor‐based approach, as applied in this study, presents advantages compared to the distribution‐based methods, but it cannot account for other sources of variability influencing the MCID calculation [21]. An appropriate evaluation of all aspects influencing the MCID should be performed to build a ‘tailored’ MCID calculation method, with clear guidelines and a common language to optimize its use in scientific literature. Among the potential influencing factors, the baseline clinical status has been suggested to play a key role [20]. Patients with poor baseline clinical conditions could present higher MCID values since they need greater improvements to perceive a real clinical benefit after being treated. On the flip side, patients in good baseline clinical conditions could consider themselves satisfied even with small improvements [7, 16, 19]. The proper identification of baseline‐related MCID values could present some methodological challenges, and Terluin et al. warned against using specific calculation methods to avoid biased results when stratifying based on the baseline score of the considered PROM in the MCID calculation [20]. Among the solutions to overcome this issue, the authors proposed a method based on the use of a second PROM that is correlated with the PROM of interest. Accordingly, the KOOS ADL subscale was chosen in the present study as a second PROM, showing a high positive correlation with the IKDC subjective score. The large size of the study population and the high positive correlation rate between the two PROMs allowed to overcome the risk of bias cautioned by Terluin et al. [20], and thus to reliably analyze how the baseline value of the IKDC subjective score influence the MCID in this series of patients affected by knee OA.

This study demonstrated that the baseline clinical status of the patients' population influenced the calculation of the MCID threshold value, with different baseline IKDC subjective scores determining different MCID values. The calculation of MCID threshold values in a population of knee OA patients treated with PRP injections allowed to identify an important heterogeneity based on the baseline clinical status. To perform this analysis, patients were first classified into clusters based on the IKDC subjective score baseline values and the MCID was calculated with an anchor‐based approach for each cluster. This analysis obtained a wide range of MCID values, from −3.1 to 16.2. Interestingly, a similarity in MCID values was found among patients in adjacent clusters by examining these values. This allowed for grouping patients into three macro clusters with similar MCID values, with consequent advantages for the sake of an easier clinical applicability. Patients with baseline IKDC subjective score <40 reported MCID values ranging between 13.5 and 16.2, patients with baseline IKDC subjective score between 40 and 69 reported MCID values ranging between 6.0 and 8.1, patients with baseline IKDC subjective score ≥70 reported MCID values ranging between −3.1 and −2.7. These findings based on the stratification of patients in relation to their clinical status confirmed the remarkable impact that baseline values of a given PROM have on the MCID quantification.

A further relevant insight derived from this analysis is that the MCID calculation based on patients with a better clinical status (IKDC subjective score ≥70) is questionable, leading to negative values of MCID. This result is clinically contradictory, as it reflects a counterintuitive situation where patients can reach the MCID after treatment even when reporting a lower score in the PROM of interest at the given follow‐up time compared to baseline, and thus being actually worsened. Therefore, the use of negative MCID values could lead researchers/physicians to misjudge the clinical results obtained, overestimating the real treatment success [8]. This highlights a critical aspect of the calculation of MCID in patients in good clinical conditions, questioning its application in these patients. Thus, when calculating the MCID in relation to a given PROM, a cut‐off value for the baseline score should be identified, excluding from the MCID calculation patients with too high baseline clinical values to avoid the risk of misinterpreting the real potential of the delivered treatment.

MCID can be used instead with patients with poorer baseline clinical conditions. However, also in this case, this study underlined the importance of critically analysing the patient cohort investigated, as two main macro clusters were identified. Patients with IKDC <40 obtained a mean MCID value of 14.6, while patients with baseline IKDC subjective score between 40 and 69 obtained a mean MCID value of 7.2. This translates into clinical practice as a different perception by patients of the clinical improvement after treatment based on their baseline clinical condition. In fact, patients with the lowest baseline clinical score needed a greater improvement to perceive a real clinical benefit after receiving a specific treatment, while patients in better clinical conditions could perceive a benefit even from a smaller clinical improvement. Patients' expectations and satisfaction could be highly influenced by the baseline conditions, as well as the interpretation of study results.

This study underlined new crucial aspects to improve the use of MCID, but it still presents some limitations. In the present work, the stratification of the study population in relation to the baseline values of the IKDC subjective score led to heterogeneous groups' size in terms of the number of patients. In future studies, larger cohorts of patients should be evaluated to have equally numerous groups of patients after stratification. Moreover, future studies should explore if the same findings can be obtained with other series, other treatments, as well as other PROMs and follow‐up times. Nevertheless, this analysis allowed us to obtain new important insights on the influence of baseline clinical conditions on the MCID calculation. MCID is a useful parameter to interpret the results of clinical trials, but caution is needed when using this tool and a careful evaluation of the baseline scores of the evaluated PROM should be performed. Patients in baseline good clinical conditions should not be considered when calculating the MCID in order not to obtain too low or negative values, avoiding the risk of overestimating the effectiveness of the evaluated treatment. Moreover, patients should be stratified at least into macro clusters, to properly define the clinically relevant improvement. These factors should be considered when drafting common guidelines for MCID calculation and use in both clinical practice and research settings.

## CONCLUSIONS

This study demonstrated that the baseline patient clinical status influences the improvement needed to be perceived as clinically relevant. Patients with a worse baseline clinical status presented higher MCID levels, while MCID lost significance in patients with high baseline clinical values. These findings warrant applying general thresholds to a patients' cohort, showing the remarkable impact of the baseline clinical status. Patient stratification ensures a proper quantification of MCID values and the identification of patients benefiting from the studied treatment.

## AUTHOR CONTRIBUTIONS

**Giuseppe Filardo**: Conceptualization; writing—review and editing; supervision. **Luca Andriolo**: Methodology; writing—review and editing. **Elettra Pignotti**: Methodology. **Alessandro Di Martino**: Methodology. **Marco Franceschini**: Data curation; writing—original draft preparation. **Angelo Boffa**: Writing—original draft preparation. **Stefano Zaffagnini**: Supervision. All authors have read and agreed to the published version of the manuscript.

## CONFLICT OF INTEREST STATEMENT

Stefano Zaffagnini reports nonfinancial support from personal fees from I+SRL and grants from Fidia Farmaceutici SPA, Cartiheal Ltd., IGEA Clinical Biophysics, Biomet and Kensey Nash, outside the submitted work. The remaining authors declare no conflicts of interest.

## ETHICS STATEMENT

Institutional review board approval Prot. no. 0015664 at the Rizzoli Orthopedic Institute (Bologna, Italy). Obtained at the time of patients' enrolment.

## Data Availability

The data have been reported in the manuscript. Additional data can be requested from the corresponding author.
